# Effectiveness and Safety of Intracavernosal IncobotulinumtoxinA (Xeomin^®^) 100 U as an Add-on Therapy to Standard Pharmacological Treatment for Difficult-to-Treat Erectile Dysfunction: A Case Series

**DOI:** 10.3390/toxins14040286

**Published:** 2022-04-16

**Authors:** Francois Giuliano, Pierre Denys, Charles Joussain

**Affiliations:** 1 Neuro-Uro-Andrology R. Poincare University Hospital, AP-HP, 104 Bvd R. Poincare, 92380 Garches, France; pierre.denys@aphp.fr (P.D.); charles.joussain@uvsq.fr (C.J.); 2 Faculty of Medicine, Versailles Saint Quentin University, Paris Saclay, 78180 Montigny le Bretonneux, France

**Keywords:** botulinum toxin A, combination therapy, non-responders, erectile dysfunction, pharmacology

## Abstract

Registered pharmacological treatments are insufficiently effective for erectile dysfunction (ED) in around 30% of affected men. Intracavernosal injection (ICI) of ona- and abobotulinumtoxinA can reduce ED in insufficient responders. We aimed to assess the safety and effectiveness of incobotulinumtoxinA ICI as an add-on therapy to phosphodiesterase-type 5 inhibitors (PDE5-Is) or prostaglandinE1 ICIs (PGE1 ICIs) to treat ED that did not respond sufficiently to this treatment alone. We retrospectively analyzed data from 66 men with difficult to treat ED treated with single or repeated incobotulinumtoxinA 100U ICI as an add-on therapy. Response rate (increase in International Index of Erectile Function-Erectile Function domain score ≥ the minimum clinically important difference) was 52% (median (1st–3rd quartile) 43.5 (34–71) days post-incobotulinumtoxinA ICI). ED etiology (except spinal cord injury) and severity did not influence effectiveness. Only a clinically significant response to the 1st injection predicted a request for a 2nd injection (OR = 5.6, 95%, CI 1.6–19.4). Three men reported mild penile pain during the injection. These results provide preliminary evidence for the effectiveness and safety of incobotulinumtoxinA ICI as an add-on therapy to treat ED that is insufficiently responsive to standard care and provides support for the multicenter randomized clinical trial NCT05196308.

## 1. Introduction

Botulinum neurotoxins (BoNTs) administered in controlled doses can provide effective treatment for a variety of conditions [1]. Subtype A, i.e., botulinumtoxinA (BoNT/A) has the longest duration of action in humans [2] and has been approved for the treatment of strabismus, blepharospasm, muscular dystonias, hyperhidrosis, and migraine. It has also been approved as a second-line treatment for urinary incontinence caused by neurogenic detrusor overactivity and for idiopathic overactive bladder [3].

Intravesical BoNT/A injections reduce contractions of the detrusor muscle of the urinary bladder by blocking stimulus-induced acetylcholine (ACh) release from presynaptic parasympathetic terminals at peripheral neuromuscular junctions. After binding to specific membrane acceptors, BoNT/A is internalized via endocytosis into nerve terminals. The BoNT/A light chain is then translocated into the cytosolic compartment where it cleaves SNAP25, one of three essential proteins of the Soluble *N*-ethylmaleimide-Sensitive Factor Attachment Proteins (SNAP) REceptor (SNARE) complex involved in the exocytotic machinery, thus preventing the fusion of neurotransmitter-carrying vesicles with the plasma membrane of peripheral neurons and inhibiting ACh release [4].

For penile erection to occur in response to sexual stimulation, sympathetic innervation of the erectile tissue and its arterial supply must be inhibited; this allows the penile smooth muscle to relax and blood to fill in the penis. Parasympathetic activity, which controls the vasodilation of the penile arteries, can only successfully fill the penis if it follows, or at least accompanies, the initial inhibition of sympathetic activity [5]. Depression of noradrenergic transmission in the post-ganglionic nerve terminals of the sympathetic nervous system that supplies the smooth muscle was reported five decades ago [6]. Intramural BoNT/A injection throughout the whole bladder cleaves SNAP25 in adrenergic sympathetic neurons [7]. Accordingly, it has been hypothesized that intracavernosal delivery of BoNT/A could produce a transient sympathectomy within the erectile tissue [8]. Interestingly, NO (the main neurotransmitter for penile erection) released from both parasympathetic neurons and endothelial cells within the erectile tissue on sexual stimulation occurs independently from SNARE-mediated exocytosis [9].

Phosphodiesterase type 5 inhibitors (PDE5-Is) enhance the pro-erectile nitric oxide-cyclic guanosine monophosphate (NO-cGMP) signaling pathway that is responsible for the relaxation of the cavernosal smooth muscle [10]. Treatment of ED with PDE5-Is (first-line treatment) is effective in 60 to 70% of men [10]. Second-line pharmacological therapy consists of self intracavernosal injections of prostaglandinE1 (PGE1 ICIs) [11]; this treatment has a 76% efficacy rate when flexible dosing techniques are used (range 2.5–30 μg) [12]. Thus, there is a need for effective treatment for the proportion of men with ED who do not respond sufficiently to these standard treatments.

A growing body of evidence suggests that off-label BoNT/A intracavernosal injections (ICI) can improve erectile function in ED that responds insufficiently to registered pharmacological treatments. The results of preliminary studies suggest that two BoNT/A products, onabotulinumtoxinA (Botox^®^; Allergan Inc., Irvine, CA, USA) and abobotulinumtoxinA (Dysport^®^; Ipsen, Slough, UK/Galderma, Paris, France) may effectively treat this condition [13,14,15,16,17]. OnabotulinumtoxinA and abobotulinumtoxinA formulations contain the neurotoxin as part of a larger protein complex with complexing (accessory) proteins that are not required for the pharmacological activity of the neurotoxin. IncobotulinumtoxinA (Xeomin^®^; Merz Pharmaceuticals GmbH, Frankfurt, Germany) is a third registered BoNT/A formulation, free from complexing proteins [18].

The primary aim of this exploratory study was to assess the safety and effectiveness of incobotulinumtoxinA ICI as an add-on therapy to PDE5-Is or PGE1 ICIs to treat ED that did not respond sufficiently to this treatment alone; the secondary aim was to determine if ED severity, etiology, or risk factors predicted the response to this treatment.

## 2. Results

**Figure 1:**
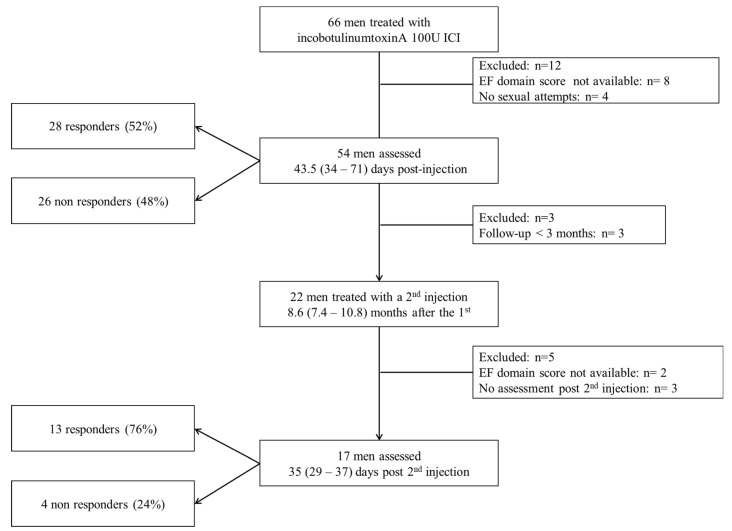
Flow-chart of men treated with incobotulinumtoxinA IC 100U. Responders and non-responders were defined according to [19].

Sixty-six men received at least one incobotulinumtoxinA ICI. The (erectile function) EF domain score, post-injection, was not available for 8 men, and 4 could not be assessed because of a lack of sexual attempts post-injection; therefore, 54 men were included (Figure 1).

### 2.1. Response to the First IncobotulinumtoxinA ICI

The mean (SD) age was 56.6 (14.3) years (range: 29–88) and the median duration of ED was 3.5 (2–9.3) years. The ED risk factors and etiologies for the 54 men who responded insufficiently to PDE5-Is or prostaglandinE1 (PGE1) ICI and were treated with incobotulinumtoxinA IC 100U were as follows: cardio-metabolic including diabetes, spinal cord injury, post-radical prostatectomy or “no identified organic risk factor/etiology or other comorbidity” in respectively 25 (46%), 21 (39%), 14 (26%) and 6 (11%). The total is >100% because some participants had >one risk factor and/or etiology.

The treatments for which the response was insufficient were PDE5-Is highest approved dose on demand (sildenafil 100 mg, vardenafil 20 mg, or tadalafil 20 mg) or daily (tadalafil 5 mg) for 43/54 men (80%) and/or PGE1 ICI (mean (SD) dose 42 (20) µg for 12/54 men (22%)). The median baseline EF domain score on treatment, i.e., prior to incobotulinumtoxinA ICI was 13 (8–19). The severity of ED on pharmacological treatment prior to incobotulinumtoxinA ICI according to EF-domain score [19] in the 54 participants was mild, moderate, and severe in respectively 22 (41%), 13 (24%), and 19 (35%) men.

**Figure 2:**
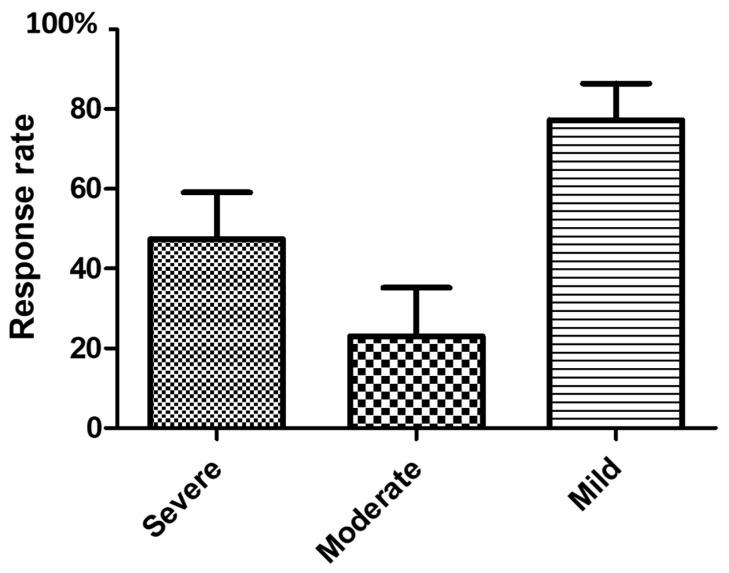
Response rates in 54 men (achievement of a clinically relevant improvement of erectile function [19] on pharmacological treatment at the first follow-up visit median (1st quartile–3rd quartile) 43.5 (34–71) days post-incobotulinumtoxinA ICI according to ED severity based on the EF domain score: mild = 17–25 points, moderate = 11–16 points and severe = 6–10 points [19].

The first follow-up assessment was performed 43.5 (34–71) days post-injection. EF-domain score post-injection was 21 (12–26). A total of 28/54 (52%) men responded: their EF domain score was 26 (22–29), corresponding to an increase of 8 (6–15) points from baseline. Figure 2 presents response rates according to ED severity. ED severity was not associated with the response to incobotulinumtoxinA ICI according to the univariate logistic regression.

**Figure 3:**
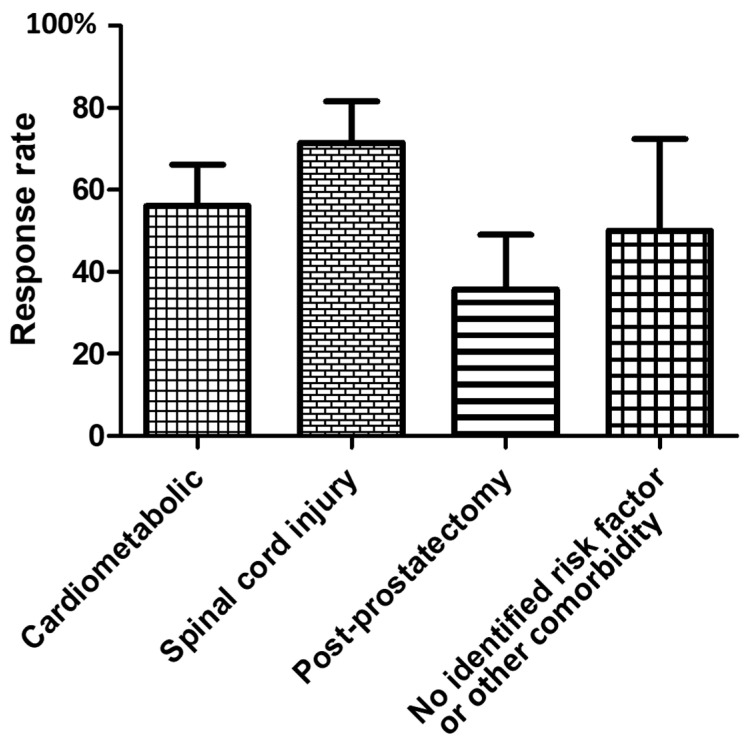
Response rates in 54 men (achievement of a clinically relevant improvement of erectile function [19] on pharmacological treatment at the 1st follow-up visit median (1st quartile–3rd quartile) 43.5 (34–71) days post-incobotulinumtoxinA ICI according to ED risk factors and etiology(ies). The cardio-metabolic etiology includes diabetes mellitus.

Figure 3 presents response rates according to ED risk factors and etiology(ies). The univariate logistic regression identified spinal cord injury as the only factor associated with response to incobotulinumtoxinA ICI (OR = 3.4, 95% CI 1.1–11.0).

### 2.2. Second IncobotulinumtoxinA ICI

In total, 22 of the 51 (43%) men who were followed for more than 3 months requested a 2nd injection that was administered 8.6 (7.4–10.8) months after the 1st (Figure 1). Among these, 5 requested the 2nd injection despite not having responded to the 1st. Table 1 shows the characteristics of men who requested or did not request a 2nd injection.

The univariate logistic regression identified a positive response to the 1st injection as the only factor associated with requesting a 2nd injection (OR = 5.6, 95%, CI 1.6–19.4).

The EF domain score was missing for 2 men at the time of the 2nd injection. Three men were not assessed after the 2nd injection. For the 17 men who were assessed at the time of the 2nd injection, the EF score was 18 (16–21) points and increased to 27 (21–29) points 35 (29–37) days post 2nd injection (their EF score at baseline was 17 [12,13,14,15,16,17,18,19,20,21] points). After the 2nd injection, 13/17 (76%) men responded compared to baseline. Of these, 11 had responded to the 1st injection. Two responders to the 1st injection were no longer responders after the 2nd. Among the 13 responders to the 2nd injection, 7 had mild, 2 moderate, and 4 severe ED on treatment at baseline. Six men had cardiometabolic comorbidities, 8 had spinal cord injury, 2 were post-prostatectomy, and 2 had other or no comorbidities.

### 2.3. Further IncobotulinumtoxinA ICI

At the time of writing, 5 men had requested a 3rd injection which was administered 8 (6.2–8.4) months after the previous one. Two were not assessed at the time of the 3rd injection. The EF domain score of the 3 men who were assessed at the time of the 3rd injection was 20, 23, and 29 points. EF domain score was re-assessed 35, 54, and 32 days after the 3rd injection. EF score increased to 28, 24, and 30 points from 8, 22, and 21 points at baseline, respectively. All 3 men responded, 2 had mild and 1 had severe ED on treatment at baseline. Two had spinal cord injury, and 1 had no comorbidity. These 3 men had all responded to the 1st injection.

One man received a total of 5 injections with a delay of 6.7 months between the 3rd and the 4th injections and 2.7 months between the 4th and the 5th injections. EF score was assessed respectively 46 and 49 days after the 4th and the 5th injections. After both injections, the EF domain score increased from 20 to 28 points. This man had severe ED (EF domain score 8) at baseline due to spinal cord injury (SCI).

The median delay between injections for men who received at least 2 injections (29 repeated injections in total) was 8.2 (7–10) months.

### 2.4. Men Who Did Not Request Further IncobotulinumtoxinA ICI

Despite a clinically significant improvement 42 (29–50) days following the 1st incobotulinumtoxinA ICI, 11 men did not request a 2nd injection. Of these, 1 was lost to follow-up, 2 were no longer sexually active, and 1 switched to a vacuum device because of lack of effectiveness. Among the 7 who were re-assessed, EF domain score was 23 (20–28) points, 10.8 (8.4–13.9) months post-injection and their baseline EF-domain score at baseline on pharmacological treatment was 17 (13–18.5) points; 5/7 remained responders to incobotulinumtoxinA ICI.

### 2.5. Reported Side Effects of IncobotulinumtoxinA ICI

Three men reported pain during one injection out of the 83 injections performed. The pain was mild and transient, and analgesia was not required.

## 3. Discussion

This study is the first to provide evidence of the safety and effectiveness of single or repeated incobotulinumtoxinA ICIs for the treatment of ED in men with an insufficient response to registered pharmacological treatment.

The 3 BoNT/A formulations approved worldwide for a variety of indications (inco- ona- and abo-botulinumtoxinA) have the same mechanism of action. IncobotulinumtoxinA has a similar efficacy to onabotulinumtoxinA with a comparable adverse event profile when a clinical conversion ratio of 1:1 or 1:1.2 is used [20]. Clinical data are consistent with preclinical comparability data [Scaglione]. Since the potency of Botox^®^ and Xeomin^®^ is identical, they can be compared using a 1:1 conversion ratio [21]. Studies have repeatedly reported the effectiveness of a 100U dose of onabotulinumtoxinA in difficult-to-treat ED [14,16,17]; therefore, we used the same dose of incobotulinumtoxinA in the present study. The conversion ratio between onabotulinumtoxinA (or incobotulinumtoxinA) and abobotulinumtoxinA is more debated [20]. The most commonly reported conversion ratios are 1:3 and 1:4 [22].

Our results confirm previous reports regarding the efficacy of ona- and abo-botulinumtoxinA ICI as an add-on therapy to registered treatments for difficult-to-treat ED [15,16]. It is noteworthy that across these studies, which involved comparable samples of men with ED, the efficacy of the three formulations was similar (taking into account the conversion ratios). Indeed, the response rate at the 1st post-injection follow-up visit was 54% with abobotulinumtoxinA 250 or 500 U Speywood in one study [15], 50% with ona- 100 U or abo-botulinumtoxinA 250 or 500 U Speywood in another study [16] and 52% with incobotulinumtoxinA 100 U in the present study. A significant improvement in erectile function was also reported for difficult-to-treat ED following onabotulinumtoxinA 50 and 100 U ICI alone [13,14,17]. Nevertheless, when BoNT/A is not combined with registered pharmacological treatments, the magnitude of the effect on ED is smaller [13,14,17].

These results raise the question of the best therapeutic paradigm for the use of BoNT/A ICI in insufficient responders to registered pharmacological treatments. An experimental study in an animal model of vasculogenic ED showed a synergistic effect of BoNT/A ICI combined with PDE5I [23]. Although caution should be taken when extrapolating experimental results to the clinical situation, these results support the combined administration of BoNT/A ICI and PDE5Is to optimize treatment effectiveness. Furthermore, this combined treatment is supported by the proposed mechanism of action of BoNT/A ICI. Animal studies have suggested that BoNT/A ICI could facilitate erection by inhibiting norepinephrine (NE) release from cavernosal sympathetic nerve terminals: the release of NE from sympathetic vasoconstrictor neurons was decreased in vitro by BoNT/A [24]. In adult rats, BoNT/A ICI increased the resting diameter of the sinusoids of the corpora cavernosa [13] and in vivo injection of BoNT/A into the urethra inhibited the release NE from the urethra [25]). Moreover, when combined with sildenafil, guanethidine ICI exerts a synergistic pro-erectile effect in anesthetized rats: guanethidine ICI depletes tissue stores of NE and decreases reuptake of NE, thereby lowering sympathetic tone [26].

The consistent results found by the present study and previous clinical studies that used other types of BoNT/A support the use of BoNT/A ICI for the treatment of insufficient responders to PDE5-Is. One-third of men with ED of all etiologies do not respond sufficiently to PDE5-Is [10,27], thus, these results have wide clinical implications. Interestingly, neither ED severity nor its etiology or risk factors, apart from spinal cord injury, predicted the effectiveness of incobotulinumtoxinA ICI. These results suggest that incobotulinumtoxinA ICI add-on therapy could be used to treat ED of all severities and etiologies. Sympathetic overactivity has been reported in the cavernosal tissue in post-radical prostatectomy patients [28] as well in men with psychogenic ED [29]. Furthermore, the increased sympathetic tone is a common pathophysiological feature in cardiovascular diseases [30]. The effect of incobotulinumtoxinA ICI may therefore not be specific to a particular etiology. Sympathetic innervation of the erectile tissue originates from the sympathetic centers located in the T12-L2 spinal segments. Sympathetic innervation of the lower urinary tract originates from the same centers. In men with spinal cord injury, sympathetic overactivity can cause detrusor-sphincter dyssynergia [31]. The presence of detrusor-sphincter dyssynergia in men with spinal cord injury who respond to incobotulinumtoxinA has not yet been evaluated: if present, dyssynergia could explain the positive response to the treatment.

The present study also provides preliminary information regarding the effectiveness of repeated incobotulinumtoxinA ICI. Of note, the only factor that predicted a request for a 2nd injection was a clinically significant response to the 1st injection; this finding is clinically coherent and further highlights the clinical effectiveness of this add-on treatment. Together, these findings suggest that in clinical practice, it may be necessary to try a 1st injection and determine subsequent treatments according to the response obtained.

In the detrusor muscle, BoNT/A inhibits acetylcholine release by parasympathetic neural terminations [32]. A 3-year open-label study of intradetrusor injections of onabotulinumtoxinA for incontinence caused by neurologic detrusor overactivity found a median duration of effect of 9 months following a single injection, although the effect varied across individuals [33]. Interestingly the median delay between 2 incobotulinumtoxinA ICI in the present study was 8.2 months, which was very similar to the repeated intradetrusor injections in the previously mentioned study.

We found that incobotulinumtoxinA ICI was safe, with less than 4% with minor, local side effects and no systemic adverse events. The only local side effect was mild pain during injection, which was likely related to the injection procedure and not to the incobotulinumtoxinA.

The main limitations of this study are that it was single-center, uncontrolled, and retrospective. The lack of a control group limits the conclusions. However, in two randomized, placebo-controlled trials of BoNT/A ICI for difficult-to-treat ED, the placebo response rate was null [14,17]. The relatively high percentage of men with SCI in the sample (because our center is specialized in neurological conditions) may have biased the results.

## 4. Conclusions

The results of this exploratory study showed that approximately half of men who responded insufficiently to pharmacological treatments experienced a clinically meaningful improvement in erectile function after incobotulinumtoxinA (Xeomin^®^) 100U ICI. Furthermore, the treatment was safe. This therapeutic effect was sustained over time with repeated injections in a proportion of men. These encouraging results provide a rationale for a prospective, randomized, double-blind, placebo-controlled, multicenter study comparing the efficacy of incobotulinumtoxinA (Xeomin^®^) 100U as an add-on therapy to sildenafil 100 mg on-demand with placebo ICI combined with sildenafil 100 mg on demand for the treatment of ED that is not sufficiently responsive to standard therapy (PDE5-Is) (ClinicalTrials.gov Identifier: NCT05196308).

## 5. Methods

### 5.1. Design

We conducted a single-center, retrospective study using a database of consecutive medical records of men with ED who responded insufficiently to registered pharmacological treatments and who received single or multiple incobotulinumtoxinA (Xeomin^®^) 100 U injections between November 2019 and January 2022. The database was approved by the French Data Protection Authority (Commission Nationale Informatique et Libertés) under the registration number 2209010v0 in agreement with the French legislation for retrospective studies. Written informed consent was obtained from participants and they were informed that they could deny access to their personal and medical data at any time. All medical files were anonymized. The men did not pay for the injections, which were provided as a compassionate treatment by the hospital.

### 5.2. Participants

Men were enrolled if (i) they were aged ≥18 years, with a diagnosis of ED and a history of insufficient response to any available PDE5-I administered at the highest approved dose for at least 3 months, either on-demand or daily (tadalafil), or to PGE1 ICIs with a dose up to 60 µg, and (ii) they received single or repeated incobotulinumtoxinA ICI as an add-on therapy to their current pharmacological treatment, with at least one follow-up visit after the 1st injection.

### 5.3. Procedure

IncobotulinumtoxinA ICI was performed as previously described [15]. Briefly, an adjustable penile loop ring was placed by the physician at the penile crus prior to the injections and removed 30 min later. Two syringes equipped with a 13 mm long 29 ½ G needle were used to deliver 100 units of incobotulinumtoxinA with 50U in 0.5 mL in each corpus cavernosum. Following incobotulinumtoxinA ICI, the men were advised to attempt sexual intercourse using their usual pharmacological treatment.

Further injections of incobotulinumtoxinA were performed on request and were usually because of a decrease in efficacy of the previous injection or to gain additional improvement of erectile function. PDE5-Is or PGE1 ICIs were continued during the period of incobotulinumtoxinA ICI injections. Further injections were performed at least 3 months after the previous injection to decrease the risk of antibody formation against BoNT/A.

A 1st follow-up visit (face-to-face or by telephone) was scheduled during the 2nd-month post-injection. Men whose ED improved also underwent a 2nd visit during the 7th-month post-injection. Consultations could also be arranged anytime on request by the individual. The 1st visit of the 1st participant was in November 2019 and the last visit of the last participant was in February 2022.

### 5.4. Outcomes

The primary endpoint was the EF domain score at the 1st follow-up visit after each incobotulinumtoxinA ICI. The secondary endpoints were:

(i) the achievement of a clinically relevant improvement in erectile function at the 1st post-injection follow-up visit. The change was considered clinically relevant if it was ≥the minimally clinical important difference (MCID) for EF score corrected for baseline severity of the ED. The MCID for each level of ED severity was mild: 2 points, moderate: 5 points, and severe: 7 points [19].
(ii) the factors associated with the response to the 1st incobotulinumtoxinA ICI at the 1st follow-up visit and with the request for a 2nd injection.
(iii) patient-reported side effects.

We categorized participants according to ED etiology(ies) and/or risk factor(s), the duration of ED, their pharmacological ED treatment prior to incobotulinumtoxinA ICI and the severity of the ED, i.e., mild, moderate or severe. ED severity was determined with the International Index of Erectile Function-Erectile Function (IIEF-EF) domain score [19]: mild = 17–25 points, moderate = 11–16 points and severe = 6–10 points. The response to incobotulinumtoxinA ICI was determined by the difference between the EF domain score at the 1st follow-up visit during the 2nd-month post-injection and baseline score (i.e., before incobotulinumtoxinA injection, with PDE5-Is or PGE1 ICIs).

### 5.5. Statistical Analysis

Continuous variables were expressed as means (standard deviations). The EF domain score, time between the 1st incobotulinumtoxinA ICI, and the assessment and duration of ED were presented with medians (1st–3rd quartile). No formal sample size calculation was performed and all analyses were exploratory. Odds ratios (ORs) were calculated from logistic regressions in a univariate analysis (Stata/MP 17.0 Timberlake, Richmond upon Thames, UK) to identify factors associated with the response at the 1st post-injection follow-up visit. We included the following variables in the univariate logistic regression: age, ED duration, comorbidities, ED treatment prior to the injection, EF domain score at baseline and ED severity. To identify the factors associated with a request for a 2nd incobotulinumtoxinA ICI, we included the following variables in the univariate logistic regression: age, ED duration, comorbidities, ED treatment prior to incobotulinumtoxinA ICI, EF domain score at baseline, ED severity and response to the 1st injection.

## Figures and Tables

**Table 1:** Characteristics of insufficient responders to PDE5-Is or PGE1-ICIs treated with incobotulinumtoxinA ICI 100U as an add-on therapy and followed >3 months post-injection who did not request, and who requested, a 2nd injection.

	Men Who Did Not Request a 2nd Injection (*n* = 29)	Men Who Requested a 2nd Injection (*n* = 22)
**Age (years), mean (SD)**	59.3 (13.3)	53 (14.1)
**ED duration (years), median (1st quartile–3rd quartile)**	5 (2–6)	3 (2–12.7)
**ED severity according to ED domain score** [19]		
**Severe *n* (%)**	10 (35)	8 (36)
**Moderate *n* (%)**	7 (24)	4 (18)
**Mild *n* (%)**	12 (41)	10 (45)
**ED risk factors and etiologies**		
**No identified organic risk factor/etiology or** **other comorbidity *n* (%)**	4 (14)	2 (9)
**Cardiometabolic *n* (%)**	13 (45)	10 (45)
**Spinal cord injury *n* (%)**	8 (28)	11(50)
**Post-prostatectomy *n* (%)**	9 (31)	5 (23)
**ED treatment prior to incobotulinumtoxinA ICI**		
**PDE5-Is *n* (%)**	23 (79)	17 (77)
**PGE1 ICIs *n* (%)**	6 (21)	5 (23)
**dose PGE1 ICI (µg), mean (SD)**	43 (20)	44 (22)
**1st incobotulinumtoxinA ICI outcome**		
**EF domain score at baseline *on treatment*, median (1st quartile–3rd quartile)**	13 (7–18)	15 (8–20)
**EF domain score post 1st injection, median (1st quartile–3rd quartile)**	13 (7–23)	25 (18–29)
**Responders *n* (%)** [19]	11 (38)	17 (77)
**Time between 1st injection and 1st assessment (days), median (1st quartile–3rd quartile)**	43 (30–57)	46.5 (32–70)
